# Influences of *Serendipita indica* and *Dictyophorae echinovolvata* on the Growth and Fusarium Wilt Disease Resistance of Banana

**DOI:** 10.3390/biology11030393

**Published:** 2022-03-02

**Authors:** Chunzhen Cheng, Fan Liu, Bin Wang, Pengyan Qu, Jiapeng Liu, Yongyan Zhang, Wei Liu, Zheng Tong, Guiming Deng

**Affiliations:** 1 College of Horticulture, Shanxi Agricultural University, Jinzhong 030801, China; yanyan5235@outlook.com (P.Q.); zhyy0425@126.com (Y.Z.); wei.liu.shanxi@sxau.edu.cn (W.L.); 2 College of Horticulture, Fujian Agriculture and Forestry University, Fuzhou 350002, China; lfan1111@126.com (F.L.); wb971220@163.com (B.W.); a15555767583@163.com (J.L.); 3 Institute of Fruit Tree Research, Guangdong Academy of Agricultural Sciences/Key Laboratory of South Subtropical Fruit Biology and Genetic Resource Utilization (Ministry of Agriculture and Rural Affairs)/Guangdong Province Key Laboratory of Tropical and Subtropical Fruit Tree Research, Guangzhou 510640, China; 4 Institute of Tropical Bioscience and Biotechnology, Chinese Academy of Tropical Agricultural Sciences, Haikou 571101, China; tongzheng@itbb.org.cn

**Keywords:** Fusarium wilt disease, *Serendipita indica*, bamboo fungus, growth, banana

## Abstract

**Simple Summary:**

By using ‘Zhongjiao No.3’ and ‘Zhongjiao No.4’ banana seedlings as plant materials, we investigated the influences of *Serendipita indica* and bamboo fungus (*Dictyophorae echinovolvata*) culture substrates on the growth and wilt disease resistance of banana. *S. indica* colonization significantly enhanced the growth of the two banana cultivars, while banana seedlings grown in nutrient soil containing bamboo fungus culture substrates inoculated with *D. echinovolvata* showed suppressed growth. Moreover, both *S. indica* colonization and *D. echinovolvata* culture substrates addition can alleviate the disease symptoms caused by *Fusarium oxysporum* f. sp. *cubense* tropical race 4 (*Foc*TR4), and their combined application showed the best disease resistance enhancement effect. The results obtained in this study can provide a basis for the application of *S. indica* and bamboo fungus in the prevention and control of banana Fusarium wilt disease in the future.

**Abstract:**

Recently, many control methods have been tried and applied in the Fusarium wilt disease control of banana and have achieved definite progresses. In this study, by using ‘Zhongjiao No.3’ and ‘Zhongjiao No.4’ banana seedlings as materials, the effects of *Serendipita indica* and bamboo fungus (*Dictyophorae echinovolvata*) culture substrates on the growth and Fusarium wilt disease resistance of banana were investigated. Results showed that the plant height, leaf length, leaf width, root length and root thickness, aboveground part fresh weight, root fresh weight, and relative chlorophyll content and nitrogen content in leaves of banana seedlings colonized with *S. indica* were all greater than those of non-colonized controls, while these parameters of banana seedlings grown in nutrient soil containing *D. echinovolvata* culture substrates were significantly suppressed. Both *S. indica* non-colonized and colonized seedlings cultivated in nutrient containing 1/4 *D. echinovolvata* culture substrates showed much milder symptoms compared with those cultivated in normal nutrient soil, indicating that the addition of bamboo fungus substrates to the soil can enhance the Fusarium wilt resistance of banana. The results obtained in this study can provide a basis for the application of *S. indica* and bamboo fungus in the prevention and control of banana Fusarium wilt disease.

## 1. Introduction

*Serendipita indica* (named formerly as *Piriformospora indica*) is an arbuscular mycorrhizal (AM)-like fungus isolated from the shrub roots in a desert of India by Verma et al. [1,2]. The fungus is artificially culturable and its host range is known to be one of the widest [3]. *S. indica* associations with host plants promote growth and development as well as nutrient absorption and enhanced stress resistance of host plants [3]. It has been successfully applied in banana and showed significant promoting effects on banana growth and stress resistance. *S. indica* was first applied to bananas by Madaan et al. [4], and its colonization significantly promoted the growth and yield of ‘Grand Naine’ banana (*Musa indica* cv. Grand Naine) [4]. Later, it was reported that the inoculation of *S. indica* significantly promoted the rooting and photosynthetic pigment synthesis and greatly accelerated the growth of tissue cultured ‘Tianbaojiao’ banana plantlets [5]. In addition to the growth promoting effect, *S. indica* can also enhance the resistance of banana to both abiotic and biotic stresses. For example, Li et al. reported that colonization of *S. indica* improved the banana cold resistance by stimulating antioxidant capacity, improving SS accumulation, and by regulating the expression of cold-responsive genes in banana leaves [6]; Bodjrenou et al. found that colonization of *S. indica* improved the thermal resistance of banana [7]; Cheng et al. reported that the fungus could improve the resistance of banana to *Fusarium oxysporum* f. sp. *cubense* tropical race 4 (*Foc*TR4) by regulating the antioxidant enzyme activities of banana root [8]. All this evidence indicated that *S. indica* has great potential to be applied as a production and resistance improvement agent in the banana industry.

Bamboo fungus (named as ‘Zhusun’ (ZS) in Chinese), known as ‘the queen of fungi’ and ‘the king of delicacies’, is a kind of edible mushroom in the *Dictyophora* genus with high nutritive values, immunostimulatory activity [9,10], and noteworthy anti-oxidant and anti-microbial activities [11]. In the Nanping city of Fujian province, China, people are used to adding bamboo fungus pilei to meat soup for the purpose of preventing spoilage. In one of our previous studies, we found that the crude extracts of *Dictyophorae echinovolvata* pilei, volva, and stipes all showed certain inhibitory effects on the growth of *Foc*TR4 on the PDA medium, and the pilei crude extract showed the best effect [12], suggesting that bamboo fungus has potential to be used in the agricultural control of banana Fusarium wilt disease.

Banana is an important fruit and food crop in the world [13]. As a characteristic fruit, banana has a huge development prospect and has become an important agricultural pillar in the southern subtropical region of China [14]. However, in recent years, the banana industry has suffered greatly from the devastating Fusarium wilt disease. For the environment-friendly and green prevention and control of the daunting crisis for the banana industry, numerous intensive agricultural and biological control strategies have been trialed and achieved remarkable progresses [8,15,16,17]. Given the disease resistance enhancement ability of *S. indica* and the anti-microbial activity of bamboo fungus, in this study we investigated the effects of *S. indica* colonization and *D. echinovolvata* culture substrates addition into the nutrient soil on the growth and Fusarium wilt resistance of two banana cultivars, ‘Zhongjiao No.3’ (ZJ3) and ‘Zhongjiao No.4’ (ZJ4). Growth-related parameters, including plant height, leaf length, leaf width, root length and root thickness, aboveground part fresh weight, root fresh weight, relative chlorophyll content and nitrogen content in leaves of banana seedlings colonized and non-colonized with *S. indica* (CK and S+ groups), and banana seedlings grown in nutrient soil containing different volume ratios of bamboo fungus culture substrates were measured and compared. Moreover, CK and S+ seedlings cultured in nutrient soil containing no and 1/4 bamboo fungus culture substrates were subjected to *Foc*TR4 inoculation experiment to show their influences on the appearance and development of the Fusarium wilt disease symptoms. The results obtained in this study can provide a basis for the application of *S. indica* and bamboo fungus in the prevention and control of banana Fusarium wilt disease in the future.

## 2. Materials and Methods

### 2.1. Plant and Fungi Materials

The tissue-cultured ‘Zhongjiao No.3’ (ZJ3) and ‘Zhongjiao No.4’ (ZJ4) banana seedlings used in this study were kindly provided by Fruit Research Institute, Guangdong Academy of Agricultural Sciences. ZJ4 was reported to be a banana Fusarium wilt resistant banana cultivar, while ZJ3 was somewhat sensitive to banana wilt disease. The *S. indica* and *Foc*TR4 isolates used in this experiment were kept in our lab. The bamboo fungus culture substrates (containing 68% bamboo shaving, 30% rice hull, 1% urea, and 1% quicklime, pH 4.5~5.0) that have been inoculated with *Dictyophorae echinovolvata* for one month were purchased from a mushroom market in Nanping city, Fujian province of China.

### 2.2. Preparations of S. indica and FocTR4 Inoculation Solutions

Three round *S. indica* agar discs with a diameter of 5 mm were added into a triangular flask containing 100 mL PDB medium and cultured in the dark at 200 rpm at 28 °C for three days. Next, the obtained *S. indica* fermentation solution was diluted with distilled water for about 3 times and used as inoculation solution (containing approximately 60 g mycelia per liter and 1 × 10^5^ spores per milliliter) [8]. With the same method, *Foc*TR4 fermentation solution was prepared. Then, *Foc*TR4 inoculation solution were prepared according to the method described by Wang et al. [18]. Briefly, *Foc*TR4 fermentation solution was filtered using two layers of lens papers to remove mycelia, then spores were collected by centrifugation at 4000 rpm for 5 min, re-suspended in Hoagland nutrition solution, and adjusted to a final concentration of 1 × 10^7^ spores/mL.

### 2.3. S. indica Inoculation and Colonization Observation

Roots of tissue-cultured banana seedlings were slightly wounded with surgical blades, soaked in *S. indica* inoculation solution for 6 h, and transplanted into plastic breeding bags containing nutrient soils (pH 5.5~6.0, Pindstrup, Ryomgård, Denmark). To better ensure its colonization, the *S. indica* inoculation solution was watered into the soil nearby the banana roots (S+ group) for three times [8]. Seedlings treated with the same volume and equally diluted PDB were used as controls (CK group). For the detection of *S. indica* colonization, at two weeks post-inoculation, roots of S+ plants were collected and cut into 1 cm segments, soaked in 10% KOH solution for 24 h, washed with sterile water for 5 times, treated with 1% hydrochloric acid solution for 4 min, then stained using 0.05% Trypan blue staining solution for 1 min [19] and observed under a Olympus optical microscope (Tokyo, Japan).

### 2.4. Growth Indexes Measurement

To investigate the effects of *S. indica* on the growth and development of banana, seedlings with and without *S. indica* colonization were transplanted into nutrient soil. To study the effects of bamboo fungus culture substrates on banana growth and development, half of the *S. indica* colonized and non-colonized seedlings were transplanted into nutrient soil containing no (CK group), 1/4 (1/4 group), 1/2 (1/2 group), and complete (ZS group) bamboo fungus culture substrates that have been inoculated with *D. echinovolvata* for one month, respectively. Then, all the seedlings were cultured in a greenhouse at 28 °C and transplanted into flowerpots at 2 months post-transplanting (mpt). At 1, 2, and 3 mpt, growth parameters such as plant height, leaf length, and leaf width were measured. At 3 mpt, root length, root number, root thickness, and fresh weight of overground and roots were also measured. Moreover, the chlorophyll relative content and nitrogen content in banana leaves were measured using a chlorophyll meter (TYS-4N, Beijing, China) at 3 mpt.

### 2.5. FocTR4 Inoculation

Banana seedlings without and with *S. indica* colonization were divided into two groups. One group was transplanted into nutrient soil (CK and S+ groups), and the other group was transplanted into nutrient soil containing 1/4 of bamboo fungus culture substrates (1/4Z and 1/4SZ groups). All the seedlings were cultured in a greenhouse at 28 °C for one month. Then, roots of CK and 1/4Z groups were directly immersed in *Foc*TR4 inoculation solution for 24 h (F and ZF groups) [18]. Since *S. indica* can delay the appearance of Fusarium wilt symptoms [8], to accelerate symptom appearance of *S. indica* colonized banana seedling, the roots of S+ and 1/4SZ seedlings were firstly wounded using surgical blades, then washed and soaked in *Foc*TR4 inoculation solution for 24 h (SF and SZF groups). After *Foc*TR4 inoculation, seedlings were replanted back, and the *Foc*TR4 inoculation solution was watered into the soil nearby the root system. Then, they were cultured in a greenhouse at 28 °C with a photoperiod of 12 h light/12 h dark. Five weeks later, disease symptoms of each banana seedling group were observed.

### 2.6. Statistics Analysis

For each group and each treatment, at least 10 seedlings of both the two banana cultivars were used, and all the parameters were measured for at least three times. Results for the detected parameters are presented as the mean ± standard deviation (SD). All the data analyses were performed using Microsoft Excel 2019 and IBM**^®^** SPSS**^®^** Statistics 25 (IBM Corporation, Armonk, NY, USA). Difference significance analysis among different groups was performed by using Duncan’s multiple-range test at 0.05 level. GraphPad Prism v6.01 (GraphPad Software, Inc., San Diego, CA, USA) was used for figure drawing.

## 3. Results

### 3.1. S. indica Colonization Detection Results

**Figure 1:**
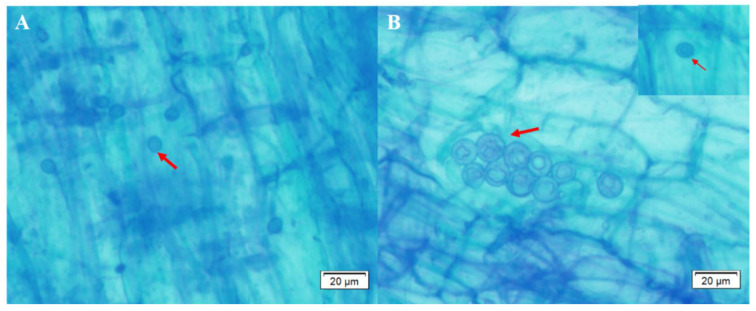
Trypan blue staining result for the observation of the *S. indica* colonization in roots of ‘Zhongjiao No. 3’ (**A**) and ‘Zhongjiao No.4’ (**B**) banana seedlings. Red arrows represent typical *S. indica* spores observed in banana roots.

Two weeks after inoculation, *S. indica* colonization in banana roots was observed (Figure 1). Typical pear-shaped spores were observed in roots of almost all the *S. indica* treated banana roots of the two banana cultivars, indicating that *S. indica* can easily colonized the roots of both ZJ3 and ZJ4 [8].

### 3.2. Effects of S. indica on the Growth of Banana Seedlings

**Figure 2:**
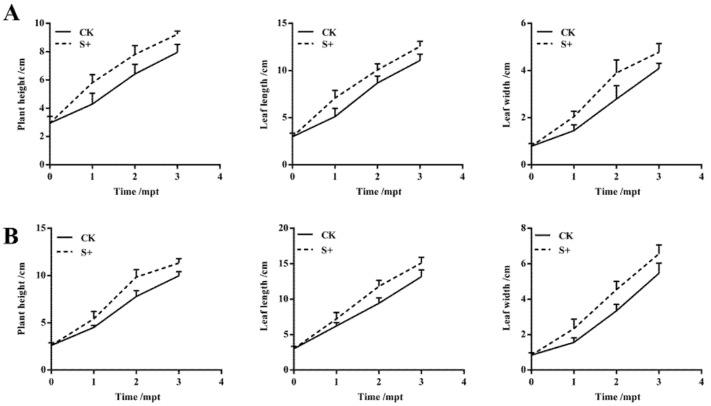
Effects of *S. indica* on the plant height, leaf length, and leaf width of ‘Zhongjiao No.3’ (**A**) and ‘Zhongjiao No.4’ (**B**) banana seedlings. CK: *S. indica* non-colonized controls; S+: *S. indica* colonized banana seedlings.

**Figure 3:**
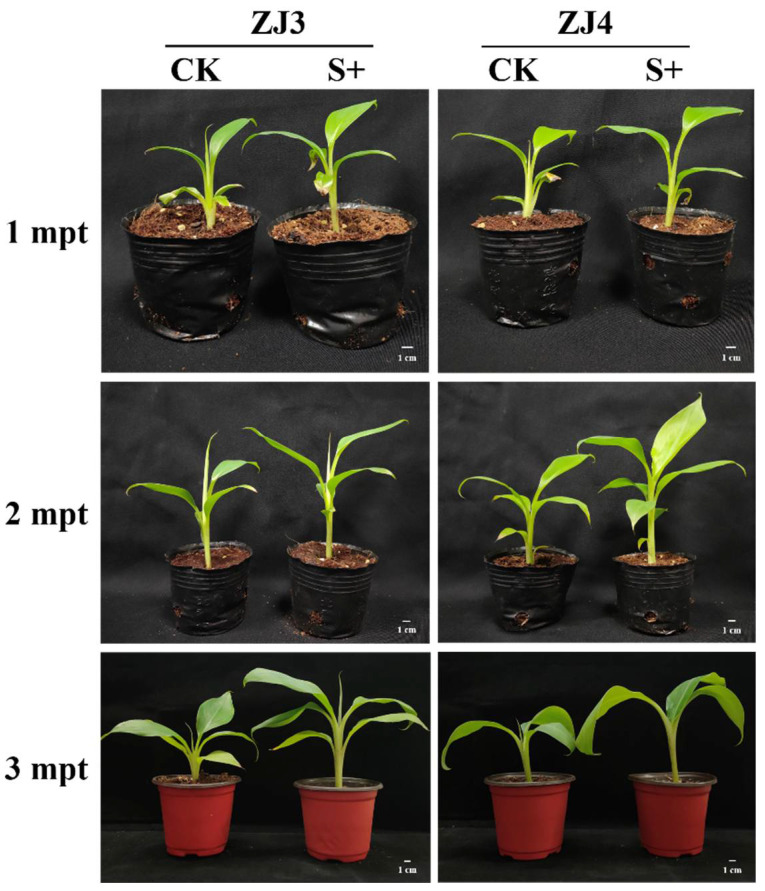
Effects of *S. indica* on the growth of ‘Zhongjiao No.3’ (ZJ3) and ‘Zhongjiao No.4’ (ZJ4) banana seedlings. CK: *S. indica* non-colonized controls; S+: *S. indica* colonized banana seedlings; mpt: months post-transplanting.

**Figure 4:**
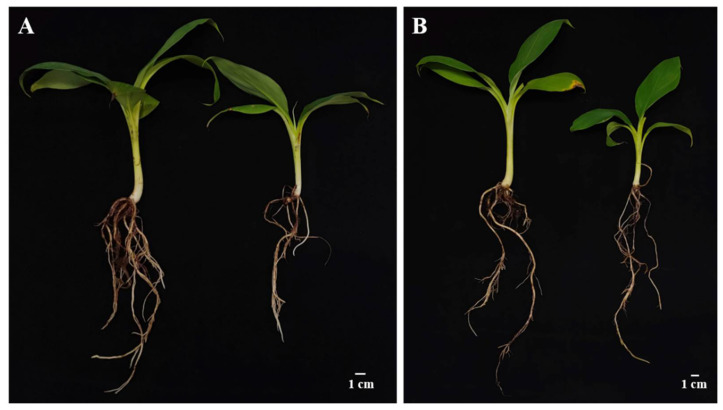
Effect of *S. indica* on the growth and root development of ‘Zhongjiao No.3’ (**A**) and ‘Zhongjiao No.4’ (**B**) banana seedlings. For both (**A**,**B**), the left one is the typical seedlings in S+ group, and the right one is typical seedlings in CK group.

After transplanting, the plant height, leaf length, and leaf width of banana seedlings were measured monthly and for a total of three times. The plant height, leaf length, and leaf width of *S. indica*-colonized ZJ3 and ZJ4 seedlings were both significantly higher than those of their corresponding control group at all the three timepoints (*p* < 0.05) (Figure 2 and Figure 3). At 1 mpt, the plant height, leaf length, and leaf width of ZJ3 seedlings in S+ group was, respectively, about 1.35-, 1.38-, and 1.41-fold of CK group (Figure 2A), and the leaf width of ZJ4 seedlings in S+ group was about 1.50-fold of CK group. At 2 mpt, the plant height and leaf length of S+ group ZJ4 was about 1.26- and 1.25-fold the CK group, respectively (Figure 2B). At 3 mpt, the root length, root thickness, aboveground part fresh weight, and root fresh weight of the two banana cultivars were measured. Almost all these parameters of banana seedlings in S+ group were significantly higher than those of the CK (*p* < 0.05) (Table 1 and Figure 4). The root fresh weight for ZJ3 and ZJ4 in S+ group was 1.72- and 1.99-fold of their corresponding CK group, respectively. The root numbers of ZJ3 and ZJ4 in S+ group were significantly greater than that in CK groups (*p* < 0.05), which was 1.47-fold and 1.27-fold of their corresponding CK, respectively.

### 3.3. Effects of S. indica on the Relative Chlorophyll Contents and Nitrogen Content in Banana Leaves

**Figure 5:**
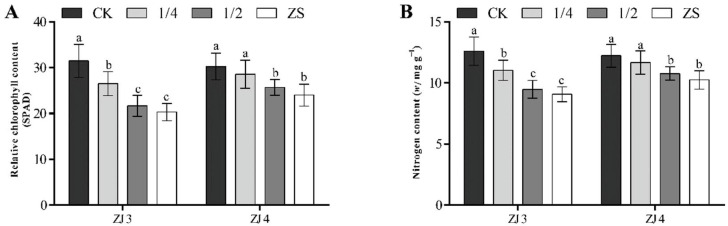
Effects of *S. indica* on relative chlorophyll content (**A**) and nitrogen content (**B**) in leaves of ‘Zhongjiao No.3’ (ZJ3) and ‘Zhongjiao No.4’ (ZJ4) banana seedlings. CK: *S. indica* non-colonized controls; S+: *S. indica* colonized banana seedlings. Different letters above the bars indicate a significant difference at 0.05 level.

At 3 mpt, the relative chlorophyll content and nitrogen content in leaves of ZJ3 and ZJ4 were measured. Results showed that both the two parameters of ZJ3 and ZJ4 in S+ group were significantly higher than those of CK (*p* < 0.05) (Figure 5). Moreover, the relative chlorophyll content and nitrogen content of ZJ4 in S+ group were both the highest, reaching 33.93 SPAD and 13.38 mg/g, respectively.

### 3.4. Effects of Bamboo Fungus on the Growth of Banana Seedlings

**Figure 6:**
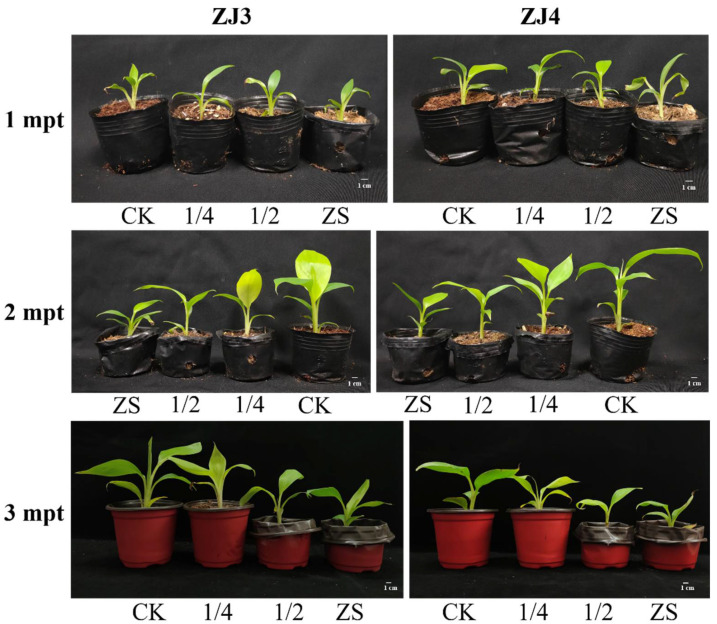
Effects of bamboo fungus on the growth of ‘Zhongjiao No.3’ (ZJ3) and ‘Zhongjiao No.4’ (ZJ4) banana seedlings. CK, 1/4, 1/2, and ZS represents typical banana seedlings grown in nutrient soil containing no, 1/4, 1/2, and complete bamboo fungus culture substrates, respectively.

The plant height, leaf length, and leaf width of banana seedlings grown in nutrient soil containing different ratios of bamboo fungus culture substrates were measured at 1, 2, and 3 mpt (Table 2 and Figure 6). At 1 mpt, no significant plant height difference was found among different groups of the two banana cultivars. There was also no significant difference in leaf width among different groups of ZJ3 seedlings, but the leaf length of ZJ3 seedlings in CK group was significantly greater than the 1/2 and ZS groups. The average leaf length and width of ZJ4 seedlings in CK group were both significantly higher than those of other groups.

At 2 mpt, the average plant height of ZJ3 seedlings followed the order CK > 1/4 > 1/2 > ZS. The leaf length and width of ZJ3 in CK group showed no significant difference with the 1/4 group, but seedlings in these two groups were both significantly higher than ZS group seedlings. The leaf length and width of ZJ4 seedlings in CK group showed no significant difference with 1/4 group, while their average plant height, leaf length, and leaf width were significantly larger than those of the ZS group. At 3 mpt, the plant height, leaf length, and leaf width of ZJ3 and ZJ4 both followed the order CK > 1/4 > 1/2 > ZS, and these parameters of CK group were all significantly higher than other groups. The plant height and leaf length of ZS group were significantly lower than those of other groups, and the leaf width of ZS group was not significantly different from that of 1/2 group, but significantly lower than that of CK group and 1/4 group.

**Figure 7:**
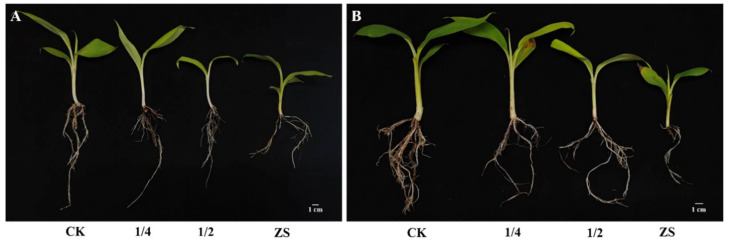
Effect of bamboo fungus on the growth and root development of ‘Zhongjiao No.3’ (**A**) and ‘Zhongjiao No.4’ (**B**). CK: seedlings grown in nutrient soil; 1/4: seedlings grown in nutrient soil containing 1/4 bamboo fungus culture substrates; 1/2: seedlings grown in nutrient soil containing 1/2 bamboo fungus culture substrates; ZS: seedlings grown in bamboo fungus culture substrates.

At 3 mpt, the biomass and root development parameters of banana seedlings were also measured (Table 3 and Figure 7). The root length, root number, root thickness, and root fresh weight of ZJ3 seedlings in CK group showed little difference with that of 1/4 group, while the aboveground part fresh weight was significantly higher than that of 1/4 group, and all indexes were significantly greater than those of 1/2 group and ZS group. Compared with the CK group, the root length, root number, aboveground part, and root fresh weight of ZJ4 seedlings in 1/4 group significantly reduced, but no significant root thickness was found between the two groups. Moreover, all the root indexes of ZS group for both ZJ3 and ZJ4 were significantly lower than those of other groups.

### 3.5. Effects of Bamboo Fungus on the Relative Chlorophyll Content and Nitrogen Content of Banana Seedlings

**Figure 8:**
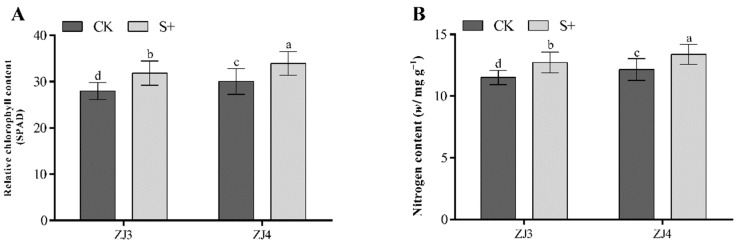
Effects of bamboo fungus on relative chlorophyll content (**A**) and nitrogen content (**B**) in leaves of ‘Zhongjiao No.3’ (ZJ3) and ‘Zhongjiao No.4’ (ZJ4) banana seedlings. CK: seedlings grown in nutrient soil; 1/4: seedlings grown in nutrient soil containing 1/4 bamboo fungus culture substrates; 1/2: seedlings grown in nutrient soil containing 1/2 bamboo fungus culture substrates; ZS: seedlings grown in bamboo fungus culture substrates. Different letters above the bars indicate a significant difference at 0.05 level.

At 3 mpt, the relative chlorophyll content and nitrogen content of banana seedlings were also measured. It was found that the relative chlorophyll content and nitrogen content of ZJ3 and ZJ4 both followed the order: CK > 1/4 > 1/2 > ZS (Figure 8). The relative chlorophyll content and nitrogen content of ZJ3 seedlings in 1/4 group was significantly lower than those of CK group seedlings, but significantly higher than 1/2 and ZS groups. There was no significant relative chlorophyll content and nitrogen content difference between 1/4 group and CK group of ZJ4, and these parameters of the two groups were both significantly larger than their corresponding 1/2 and ZS groups.

### 3.6. Effects of S. indica and Bamboo Fungus on Banana Fusarium Wilt Resistance

**Figure 9:**
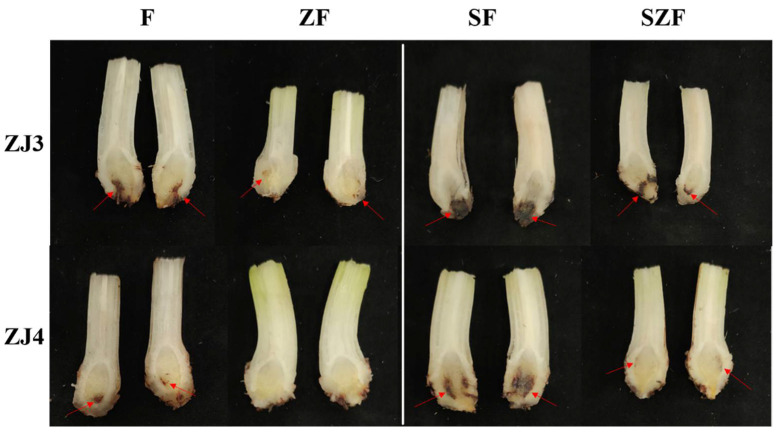
Symptoms in the corms of banana inoculated with *Foc*TR4. F, ZF, SF, and SZF represents *Foc*TR4 treated *S. indica* non-colonized control banana seedlings grown in nutrient soil, *Foc*TR4 treated *S. indica* colonized banana seedlings grown in nutrient soil, *Foc*TR4 treated *S. indica* non-colonized control banana seedlings grown in nutrient soil containing 1/4 bamboo fungus culture substrates, and *Foc*TR4 treated *S. indica* colonized banana seedlings grown in nutrient soil containing 1/4 bamboo fungus culture substrates, respectively. Red arrows represent browning areas in corms of banana.

The disease symptoms in banana were observed at 5 weeks post-pathogen inoculation (Figure 9). Results showed that the browning area in corms of ZJ3 and ZJ4 in F group accounted for about 18.53% and 9.61% of the whole corm, respectively. The ZJ3 and ZJ4 seedlings in ZF group both showed much milder symptoms than those in F groups, suggesting that the bamboo fungus culture substrates addition into nutrient soil can inhibit the infection of *Foc*TR4. To accelerate the banana wilt symptom appearance of *S. indica* colonized banana seedlings, roots were wounded before *Foc*TR4 inoculation. In SF group, the corms of ZJ3 and ZJ4 both showed a large area of browning, which accounted for about 35.50% and 27.68% of their whole corm, respectively. The symptoms in ZJ3 and ZJ4 corms in SZF group were much milder than those of SF group. This indicated that the addition of bamboo fungus culture substrates to the nutrient soil could also improve the Fusarium resistance of *S. indica* colonized banana (Figure 9). Moreover, consistent with their Fusarium wilt resistance, the symptoms in ZJ4 were found to be much milder than ZJ3.

## 4. Discussion

The banana industry has been threatened by banana wilt disease caused by *Fusarium oxysporum* f.sp. *cubense* (*Foc*) for a long time [20,21]. Up to now, however, the disease is still uncontrollable. *S. indica* is an arbuscular mycorrhizal-like fungus that can colonize plant roots extensively and form a symbiotic relationship with its host. Accumulated evidence has shown that the colonization of *S. indica* could improve the nutrient absorption, promote plant growth and development, and enhance the resistance or tolerance of banana to abiotic and biotic stresses [5,6,7,8]. Bamboo fungus is a nutritious edible mushroom which not only has high medicinal value, but also has antioxidant and anti-microbial activities. One of our previous studies had confirmed that the crude extracts of *D. echinovolvata* could inhibit the *Foc*TR4 growth on PDA medium [9]. However, whether it can be used in the control of banana Fusarium wilt disease was not explored. In this study, we applied the two fungi to banana, and investigated their influences on banana growth and *Foc*TR4 resistance. Results obtained in this study were as follows.

### 4.1. S. indica Can Promote the Growth of Banana Seedlings

*S. indica* has a growth-promoting effect similar to arbuscular mycorrhizal fungi [22]. It can colonize in the roots of various plants and increase the biomass and yield of host plants by increasing the material and nutrient absorption of host plants [23]. Inoculation of *S. indica* could advance seed germination time, promote plant height, leaf number, leaf length, leaf width, root number, and root length, and improve the quality and yield of Dendrobium officinale [24]. Cotton seedlings colonized by *S. indica* showed significantly increased plant height, stem diameter, leaf area, net photosynthetic rate, and increased drought resistance [25]. The growth promoting effects have been reported in ‘Grand Naine’ banana and ‘Tianbaojiao’ banana seedlings [4,5]. In this study, a similar promoting effect of *S. indica* in the fungal colonized ‘Zhongjiao No.3’ and ‘Zhongjiao No.4’ banana cultivars was also proved, indicating that the fungus’s growth promoting ability is not strictly cultivar-dependent. Li et al. reported that *S. indica* colonization could significantly enhance the chlorophyll accumulations in banana leaves [5]. Consistently, in this study, we also found that the fungus colonization significantly increased the relative chlorophyll content and nitrogen content of both ZJ3 and ZJ4 banana seedlings.

### 4.2. Bamboo Fungus Inhibited the Growth but Could Improve the Fusarium Wilt Resistance of Banana

Our study revealed that bamboo fungus culture substrates had a certain inhibitory effect on banana growth. By determining the growth-related parameters, we found that almost all the parameters of banana seedlings in different groups followed the order: CK > 1/4 > 1/2 > ZS. This indicated that bamboo fungus culture substrates inhibited the growth of banana seedlings, and the higher bamboo fungus culture substrates proportion in the nutrient soil, the more obvious inhibitory effect. This might be caused by the antioxidant and anti-microbial activities of *D. echinovolvata* [26]. Moreover, the nutrient content of bamboo fungus culture substrates is not as good as that of the nutrient soil and the pH value of bamboo fungus culture substrates was lower, which might also lead to its inhibitory effect on the growth of banana seedlings.

As the growth of banana seedlings grown in the nutrient soil containing 1/4 bamboo fungus culture substrates showed no significant difference compared with seedlings grown in nutrient soil at 1 mpt, we further investigated the influence of bamboo fungus culture substrates addition on the banana Fusarium wilt disease resistance. Results showed that both ‘Zhongjiao No.3’ and ‘Zhongjiao No.4’ banana seedlings grown in the nutrient soil containing 1/4 bamboo fungus culture substrates showed much milder symptoms than their control groups, indicating that bamboo fungus can alleviate the symptoms of banana Fusarium wilt to a certain extent and improve the Fusarium wilt resistance of banana. Thus, it can be concluded that the edible bamboo fungus intercropping or applying bamboo fungus fermentation solution might be a much easier and more favorable way for banana wilt disease control [27,28].

### 4.3. S. indica Improved the Fusarium Wilt Resistance of Banana and This Promoting Effect Could Be Strengthened by Bamboo Fungus Additives

*S. indica* colonization can improve the resistance of many plants to pathogenic fungi [29,30,31]. For example, evidence has shown that the *S. indica* enhanced the resistance of wheat to Fusarium head blight [32], Arabidopsis thaliana to verticillium wilt [33], tomato to early blight [34], chickpea to Botrytis cinerea [35], and so on. *Foc* is a soil-borne fungus with a long incubation time and low spore concentration [36,37], making the complete control of the banana Fusarium wilt disease very hard. A previous study had shown that *S. indica* can delay the onset of Fusarium wilt symptoms by increasing antioxidant enzyme activities [8] and by suppressing the *Foc*TR4 induced fatty acid accumulation in banana root [38]. In our present study, we conducted Fusarium wilt disease resistance evaluation experiments of *S. indica* colonized banana seedlings and compared the symptom appearance of seedlings grown in normal nutrient soil and in nutrient soil containing 1/4 bamboo fungus culture substrates. As *S. indica* colonized banana seedlings showed much delayed symptom onset, injury treatment was applied [39,40]. Results showed that, like the *S. indica* non-colonized seedlings, the symptoms in corms of *S. indica* colonized ZJ3 and ZJ4 seedlings grown in nutrient soil containing 1/4 bamboo fungus culture substrates also showed much milder symptom than those of grown in normal nutrient soil, which again indicated that bamboo fungus culture substrates addition can alleviate the Fusarium wilt symptoms and improve the resistance of banana to Fusarium wilt disease.

The development of banana Fusarium wilt epidemics could be influenced by many factors, including banana genetic resistance, soil physical and chemical properties, soil microbial populations, crop management, and so on [41,42]. The mixtures of biological control agent and organic additives have been proven to have great potential for improving plant Fusarium diseases resistance [43]. Given the growth- and stress resistance-promoting effects of *S. indica* on banana hosts and the anti-microbial activities of bamboo fungus, the application of them in banana may be considered as an eco-friendly strategy for Fusarium wilt disease control in the future [44].

## 5. Conclusions

**Figure 10:**
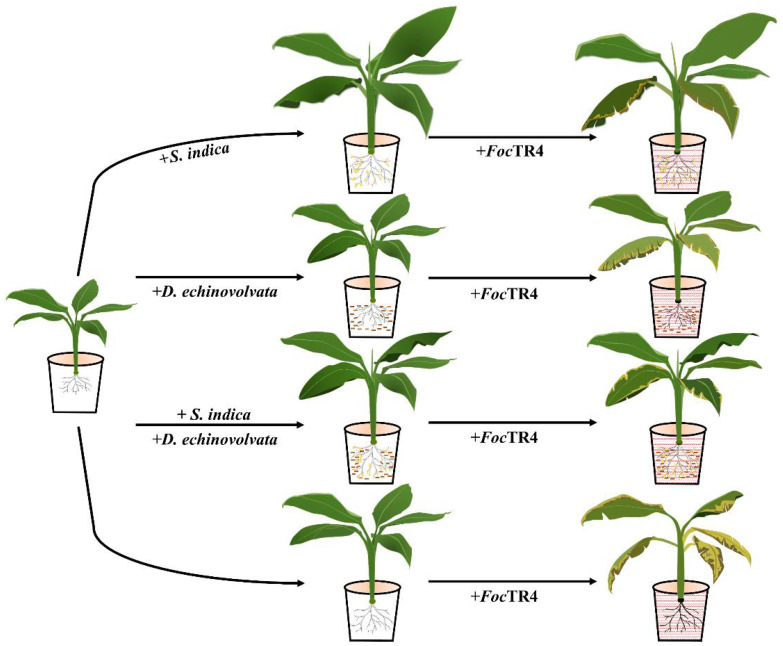
Schematic representation of the influences of *Serendipita indica* and *Dictyophorae echinovolvata* on the growth and Fusarium wilt disease resistance of banana. *S. indica* colonization (displayed in yellow) significantly enhanced the growth of banana, while banana seedlings grown in nutrient soil containing *D. echinovolvata* culture substrates (displayed in brown) showed suppressed growth. Moreover, both *S. indica* colonization and *D*. *echinovolvata* culture substrates addition alleviated the disease symptoms caused by *Fusarium oxysporum* f. sp. *cubense* tropical race 4 (*Foc*TR4, displayed in red), and their combined application showed the best effect.

Our study revealed that the *S. indica* colonization significantly improved the growth of banana seedlings, while the growth of seedlings grown in nutrient soil containing bamboo fungus culture substrates was suppressed. The suppression effect increased as the proportion of the bamboo fungus substrates increased. *S. indica* colonization could alleviate the symptoms caused by *Foc*TR4, and both the *S. indica* colonized and non-colonized banana seedlings grown in nutrient soil containing 1/4 *D. echinovolvata* culture substrates showed obvious milder symptoms compared with the seedlings grown in normal nutrient soil after *Foc*TR4 infection, indicating that the addition of *D. echinovolvata* culture substrates can alleviate the Fusarium wilt symptoms. The results obtained in this study can provide technical support and a theoretical basis for the application of *S. indica* and bamboo fungus intercropping in the prevention and control of banana Fusarium wilt disease (Figure 10). The underlying mechanism for the effects of *S. indica* and bamboo fungus on banana Fusarium wilt resistance needs to be further studied in future research.

## Figures and Tables

**Table 1:** Effects of *S. indica* on the growth indexes of ‘Zhongjiao No.3’ (ZJ3) and ‘Zhongjiao No.4’ (ZJ4) banana seedlings. CK: *S. indica* non-colonized controls; S+: *S. indica* colonized banana seedlings. The different letters in the same column represent significant difference among samples at 0.05 level.

Banana Variety	Group	Root Length/cm	Root Number	Root Thickness/mm	Aboveground Part Fresh Weight/g	Root Fresh Weight/g
ZJ3	CK	15.23 ± 0.87 d	5.67 ± 0.58 c	0.93 ± 0.02 d	2.23 ± 0.18 d	0.78 ± 0.06 d
	S+	21.73 ± 1.43 c	8.33 ± 0.58 ab	1.44 ± 0.07 c	3.58 ± 1.28 c	1.34 ± 0.13 c
ZJ4	CK	25.57 ± 1.12 b	7.33 ± 0.58 b	1.35 ± 0.05 c	4.79 ± 0.45 b	1.36 ± 0.16 c
	S+	33.67 ± 0.90 a	9.33 ± 1.15 a	1.64 ± 0.04 b	8.43 ± 0.51 a	2.70 ± 0.04 a

**Table 2:** Effects of bamboo fungus on the growth indexes of ‘Zhongjiao No.3’ (ZJ3) and ‘Zhongjiao No.4’ (ZJ4) banana seedlings. CK: seedlings grown in nutrient soil; 1/4: seedlings grown in nutrient soil containing 1/4 bamboo fungus culture substrates; 1/2: seedlings grown in nutrient soil containing 1/2 bamboo fungus culture substrates; ZS: seedlings grown in bamboo fungus culture substrates. The different letters within the same column represent significant difference among samples at 0.05 level.

Banana Variety	Group	1 mpt			2 mpt			3 mpt		
		Plant Height/cm	Leaf Length/cm	Leaf Width/cm	Plant Height/cm	Leaf Length/cm	Leaf Width/cm	Plant Height/cm	Leaf Length/cm	Leaf Width/cm
ZJ3	CK	3.53 ± 0.31 a	4.87 ± 0.32 a	1.40 ± 0.35 a	6.53 ± 0.25 a	7.57 ± 0.35 a	2.90 ± 0.26 a	8.27 ± 0.25 a	10.67 ± 0.81 a	4.60 ± 0.61 a
	1/4	3.45 ± 0.07 a	4.50 ± 0.14 ab	1.25 ± 0.07 a	5.75 ± 0.35 b	7.30 ± 0.57 a	2.30 ± 0.14 ab	7.25 ± 0.21 b	9.10 ± 0.71 b	3.45 ± 0.21 b
	1/2	3.43 ± 0.10 a	4.18 ± 0.31 b	1.15 ± 0.13 a	4.87 ± 0.31 c	6.77 ± 0.38 a	2.10 ± 0.40 bc	6.50 ± 0.79 b	8.37 ± 0.38 b	3.07 ± 0.15 bc
	ZS	3.35 ± 0.21 a	4.10 ± 0.14 b	1.15 ± 0.07 a	4.10 ± 0.28 d	5.00 ± 0.28 b	1.60 ± 0.14 c	4.70 ± 0.20 c	6.83 ± 0.35 c	2.47 ± 0.21 c
ZJ4	CK	3.68 ± 0.15 a	5.30 ± 0.46 a	1.80 ± 0.22 a	6.53 ± 0.50 a	7.53 ± 0.71 a	3.75 ± 0.37 a	8.67 ± 0.47 a	11.13 ± 0.47 a	5.93 ± 0.25 a
	1/4	3.63 ± 0.06 a	4.73 ± 0.60 b	1.40 ± 0.20 b	5.98 ± 0.56 a	6.93 ± 0.5 ab	3.23 ± 0.38 b	7.88 ± 0.54 b	10.08 ± 0.57 b	5.10 ± 0.42 b
	1/2	3.57 ± 0.40 a	4.07 ± 0.41 b	1.22 ± 0.12 b	4.98 ± 0.71 b	6.65 ± 0.62 ab	2.13 ± 0.25 c	5.83 ± 0.29 c	7.80 ± 0.30 c	3.00 ± 0.53 c
	ZS	3.50 ± 0.37 a	4.02 ± 0.32 b	1.18 ± 0.13 b	4.73 ± 0.40 b	5.83 ± 0.90 b	1.72 ± 0.10 c	4.97 ± 0.29 d	6.70 ± 0.56 d	2.70 ± 0.10 c

**Table 3:** Effects of bamboo fungus on the root growth parameters and biomass of ‘Zhongjiao No.3’ (ZJ3) and ‘Zhongjiao No.4’ (ZJ4) banana seedlings. CK: seedlings grown in nutrient soil; 1/4: seedlings grown in nutrient soil containing 1/4 bamboo fungus culture substrates; 1/2: seedlings grown in nutrient soil containing 1/2 bamboo fungus culture substrates; ZS: seedlings grown in bamboo fungus culture substrates. The different letters within the same column represent significant difference among samples at 0.05 level.

Banana Variety	Group	Root Length/cm	Root Number	Root Thickness/mm	Aboveground Part Fresh Weight/g	Root Fresh Weight/g
ZJ3	CK	20.27 ± 0.78 a	5.33 ± 0.58 a	1.10 ± 0.03 a	2.65 ± 0.42 a	0.35 ± 0.04 a
	1/4	19.30 ± 0.89 a	5.33 ± 0.58 a	1.03 ± 0.09 a	1.73 ± 0.14 b	0.30 ± 0.01 ab
	1/2	12.10 ± 1.14 b	4.33 ± 0.58 b	0.83 ± 0.08 b	1.52 ± 0.18 b	0.27 ± 0.02 b
	ZS	10.60 ± 0.75 b	4.00 ± 0.00 b	0.82 ± 0.10 b	1.49 ± 0.16 b	0.24 ± 0.04 b
ZJ4	CK	16.07 ± 0.71 a	6.67 ± 0.58 a	1.27 ± 0.04 a	4.65 ± 0.12 a	1.46 ± 0.05 a
	1/4	14.23 ± 0.47 b	5.67 ± 0.58 b	1.25 ± 0.05 a	3.77 ± 0.23 b	1.04 ± 0.07 b
	1/2	13.40 ± 0.53 b	5.33 ± 0.58 b	1.12 ± 0.05 b	1.83 ± 0.23 c	0.64 ± 0.09 c
	ZS	11.90 ± 0.40 c	4.00 ± 0.00 c	0.97 ± 0.06 c	1.91 ± 0.22 c	0.30 ± 0.06 d

## Data Availability

Not applicable.
